# Nursing Home Residents’ Preferred Recreational Activity Attendance and Depressive Symptoms Over Time

**DOI:** 10.1093/geroni/igaf122.2857

**Published:** 2025-12-31

**Authors:** Nahida Akter, Allison R Heid, Michael J Rovine, Karen Eshraghi, Katherine Abbott, Kimberly VanHaitsma

**Affiliations:** The Pennsylvania State University, University Park, Pennsylvania, United States; Independent Research Consultant, Ardmore, Pennsylvania, United States; University of Pennsylvania, Philadelphia, Pennsylvania, United States; The Pennsylvania State University, University Park, Pennsylvania, United States; Miami University, Oxford, Ohio, United States; pennsylvania state university, UNIVERSITY PARK, Pennsylvania, United States

## Abstract

Little is known about how preference-based care impacts nursing home (NH) residents’ well-being over time. The Preference Match Tracker (PMT) objectively tracks the number of recreation activities NH residents attend that match their important preferences. We explored how PMT data were linked to residents’ depressive symptoms over time. The number of preferred and non-preferred activities attended and refused to attend per week were tracked with the PMT for 586 residents over 1 year. We utilized generalized linear modeling to examine the association of preferred/non-preferred attendance/refusals for three depressive symptom groups (none, minimal, depressive symptoms). After accounting for covariates, more attendance was associated with minimal depressive symptoms over time. More total activity refusals and refusals of preferred activities were associated with minimal and mild-to-severe depressive symptoms over time and, more refusals of non-preferred activities were associated with minimal symptoms. Associations were moderated by pain, count of important preferences, cognition, and length of stay. Refusing to attend preferred activities may serve as a marker of distress. Individuals who are cognitively capable and/or living in the NH for < 90 days or experiencing pain, who are refusing preferred activities should be monitored for depressive symptoms and their recreational activity participation.

